# Environmental DNA as a Tool for the Preliminary Assessment of Vertebrate Biodiversity: A Case Study from Sicilian Freshwater Ecosystems

**DOI:** 10.3390/biology14121681

**Published:** 2025-11-26

**Authors:** Manuela Mauro, Francesco Longo, Aiti Vizzini, Mario Lo Valvo, Slobodanka Radovic, Grazia Orecchio, Rosi De Luca, Claudio Luparello, Anna Maria Mauro, Angela Cuttitta, Mirella Vazzana

**Affiliations:** 1 Dipartimento di Scienze e Tecnologie Biologiche, Chimiche e Farmaceutiche (STEBICEF), Università degli Studi di Palermo, Via Archirafi, 18, 90123 Palermo, Italyfrancesco.longo03@unipa.it (F.L.); rosi.deluca@unipa.it (R.D.L.); claudio.luparello@unipa.it (C.L.);; 2 IGA Technology Services Srl., Via Linussio, 51, 33100 Udine, Italy; 3 Arpa Sicilia UOC Acque Interne e Suolo, Lungomare Cristoforo Colombo snc, 90149 Palermo, Italy; 4 National Research Council (CNR-ISMed), Institute for Studies on the Mediterranean, Via Filippo Parlatore, 65, 90145 Palermo, Italy

**Keywords:** biodiversity, census, lakes, metabarcoding, vertebrate, Sicily

## Abstract

Freshwater ecosystems are rapidly losing biodiversity due to anthropogenic and environmental pressures. Considering that many species remain unknown, these systems represent an invaluable reservoir of biodiversity. For this reason, a prompt biodiversity census becomes essential. Environmental DNA (eDNA) has the potential to provide a faster and non-invasive alternative to traditional methods for biodiversity monitoring, conservation, and management. This study used eDNA to provide a preliminary snapshot of vertebrates in two Sicilian lakes: Lake Rosamarina and Lake Garcia. eDNA analyses identified four classes of vertebrates, 14 orders, and 16 families, with notable taxonomic differences between the two lakes. The analysis revealed the presence of several non-native and potentially invasive species, including fish (*Cyprinus carpio*, *Micropterus salmoides*, *Perca fluviatilis*, *Carassius auratus*, and *Ameiurus melas*), as well as the amphibian *Xenopus laevis* and the terrestrial mammal *Myocastor coypus*. The presence of these species, known for their ecological impact on native communities, highlights the potential of eDNA analysis as a powerful tool for the early detection and preventive biomonitoring of biological invasions. These results provide an initial assessment of vertebrate biodiversity in these sites, demonstrating how eDNA can serve as an effective method for biodiversity evaluation and monitoring.

## Simple Summary

Today, due to a variety of human and environmental impacts, we are witnessing a rapid loss of biodiversity in freshwater systems. In this context, environmental DNA (eDNA) has the potential to be a rapid and non-invasive tool for biodiversity census taking. This study used eDNA to detect vertebrates in Lake Rosamarina and Lake Garcia, identifying 4 classes, 14 orders, and 19 families. Moreover, 22 and 11 species were detected in Lake Rosamarina and Lake Garcia, respectively. These results provide a preliminary snapshot of vertebrate biodiversity in these sites, demonstrating the potential of eDNA as a tool for biodiversity census taking and monitoring.

## 1. Introduction

The ongoing decline in biodiversity across all ecosystems underscores the urgent need for robust and effective biodiversity assessment tools [1]. These assessments are critical not only for documenting and monitoring biological diversity, but also for informing conservation strategies and supporting long-term environmental management plans [2]. A growing number of species and ecosystems are under threat from climate change and the intensification of human activities [3]. Among the most vulnerable are freshwater ecosystems, which are particularly susceptible due to their distinctive biotic and abiotic characteristics [4,5]. Lakes, in particular, are exposed to a range of environmental and anthropogenic stressors—including climate change, eutrophication, hydrological modifications, excessive water abstraction, habitat degradation, salinization, acidification, and the spread of invasive species—all of which pose serious threats to their ecological integrity and biodiversity [6,7,8,9].

Traditional biodiversity assessments have predominantly relied on techniques such as visual surveys, trawling, seining, and tissue sampling [10,11]. While these methods have been widely adopted, they are often labor-intensive, costly, invasive, and potentially disruptive to ecosystems. Furthermore, they are prone to taxonomic misidentification and frequently fail to detect species occurring at low densities due to their limited capture probability [10,11].

In contrast, environmental DNA (eDNA) has recently emerged as a powerful, non-invasive tool for biodiversity monitoring. Organisms continuously release DNA into their environment through various biological materials, including skin cells, blood, saliva, sperm, mucus, eggs, feces, urine, and plant debris [12,13]. This genetic material accumulates in a wide range of environmental matrices such as soil [14], air [15], water [16], sediments [17], ice [18], and snow [19]. By targeting molecular traces released by organisms into the environment, eDNA-based approaches enable the highly sensitive detection of biodiversity across ecosystems. These methods offer clear advantages in identifying cryptic, rare, or elusive species, and are particularly effective for the early detection and monitoring of alien and invasive taxa [12,20]. As such, eDNA represents a transformative advance in ecological research and conservation biology.

In recent years, interest in eDNA as a complementary tool to traditional survey methods has increased substantially [12]. When coupled with high-throughput sequencing technologies such as metabarcoding, eDNA analysis has proven especially effective in characterizing biodiversity within ecosystems that are logistically challenging to sample. A growing body of literature supports the efficacy of eDNA for assessing biodiversity across a broad range of taxa, including aquatic, semi-aquatic, and terrestrial vertebrates [21,22,23,24,25,26,27,28,29]. The first application of eDNA to freshwater ecosystems dates back to Ficetola et al. [30], who successfully tracked the presence and movements of the invasive American bullfrog (*Lithobates catesbeianus*) in both laboratory and field conditions. This pioneering study marked a turning point in aquatic species detection, paving the way for the broader application of eDNA in ecological and environmental research. Since then, similar methodologies have been adapted for monitoring a wide range of vertebrate and macroinvertebrate species [31] across diverse environments, such as fish [32], mollusks [33], amphibians [30], reptiles [34], and mammals [35], and in their natural habitats.

The integration of eDNA into biodiversity studies could contribute to the collection of baseline ecological data, supporting a more comprehensive understanding of species distribution and ecosystem dynamics at various spatial and temporal scales [36]. However, the standardization of eDNA protocols—including sample collection, DNA capture and extraction, PCR amplification and sequencing, bioinformatics analysis, and statistical processing—is essential for maximizing the potential of this innovative technique. As biodiversity loss accelerates, the development of rapid, non-invasive, and cost-effective sampling methods such as eDNA will be crucial for detecting and conserving rare, cryptic, and threatened species while advancing global biodiversity research.

This pilot study is part of a broader project aimed at characterizing the biodiversity of inland waters in the province of Palermo, where available data remains scarce and fragmented. In a context where increasing climatic and anthropogenic pressures are driving the loss of species—which are sometimes still unknown—our work aimed to expand current information on vertebrate biodiversity in Sicilian lakes. This contribution builds upon the research line already initiated by Mauro et al. [37,38], who provided a first snapshot study of vertebrate and invertebrate biodiversity for Lake Poma, Lake Scanzano, and Lake Piana degli Albanesi. For the first time, this study focuses on assessing vertebrate biodiversity in Lake Rosamarina and Lake Garcia through the analysis of eDNA combined with high-throughput sequencing techniques (metabarcoding). These lakes are large, artificial reservoirs that play a crucial role in the inland water ecosystems of the region. Beyond their socio-economic importance for irrigation and water supply to nearby urban centers, these wetlands provide unique habitats in a landscape increasingly threatened by desertification due to climate change. Furthermore, they act as vital “oases” where numerous migratory bird species can rest and feed along one of the main migratory flyways in the Mediterranean. Despite their ecological significance, the vertebrate biodiversity of these sites has not been comprehensively assessed, nor have eDNA approaches been used, highlighting the need for studies that document and monitor these freshwater ecosystems. Regarding this, the results provide an initial picture of the vertebrate diversity present in these freshwater ecosystems, delivering baseline data that are crucial for supporting conservation strategies and guiding the sustainable management of these habitats.

## 2. Materials and Methods

### 2.1. Study Area Lakes

**Figure 1:**
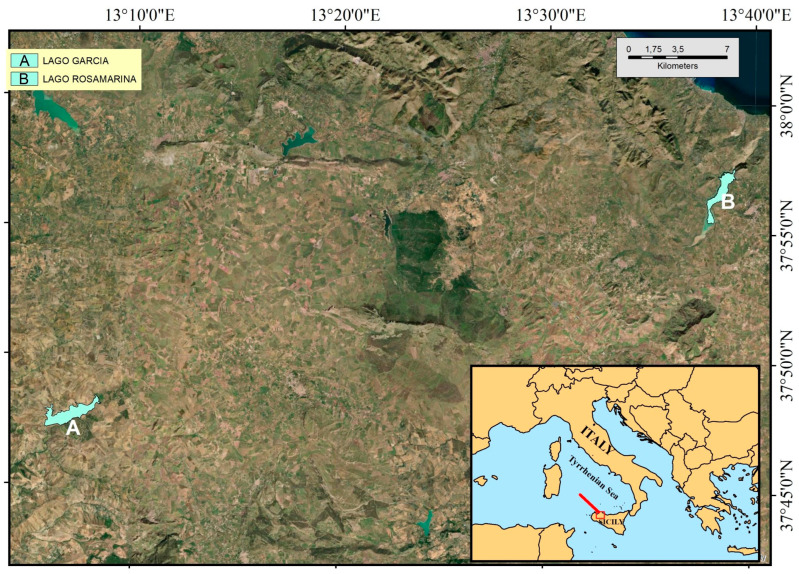
Location of the investigated lakes and coordinates of the sampling points. (A) 37°47′31.63″ N, 13°07′03.13″ E; (B) 37°56′27.27″ N, 13°38′39.48″ E.

Sampling was conducted in two artificial lakes located in northwestern Sicily: Rosamarina and Garcia, both situated in the province of Palermo (Figure 1).

The former is located in Caccamo (37°56′31.87″ N—13°38′18.67″ E), at an altitude of 176 m above sea level (asl), covering approximately 500 hectares with a perimeter of 16 km; the latter is located in Contessa Entellina (37°47′47.05″ N—13°07′13.04″ E), at an altitude of 194 m asl, with an area of approximately 507 hectares and a perimeter of about 16 km. The lakes, located about 50 km apart, are fed partly by rainwater and partly by the inflow of several rivers. They are primarily situated in agricultural areas, with the water being used both for field irrigation and as the water supply for some nearby urban centers.

### 2.2. Water Sampling and eDNA Extraction

Samples of water were collected during October 2022. This period was characterized by relatively stable climatic conditions and was a phase of increased biological activity for many aquatic organisms, allowing the effective detection of vertebrate biodiversity while minimizing potential interference from extreme weather events typical of summer or winter, as well as from anthropogenic influences. Each lake was sampled at a single point located at the intersection between the central area, the vicinity of the dam, and the external inflow. At this point, sampling was carried out in the middle of the water column. Three replicate samples were collected per point, collecting 2 L for each replicate using sterile glass bottles which had been previously autoclaved and acid-rinsed with 10% HCl. Water samples were maintained under cool, dark conditions during transportation. After arrival at the laboratory of the Department of Biological, Chemical and Pharmaceutical Sciences and Technologies (STEBICEF), water samples were vacuum-filtered under sterile conditions using HC Series vacuum filtration systems (Cheimika-HC/SLGS/F05002, Pellezzano, Italy) equipped with nitrocellulose membranes (MF-Millipore, 0.22 µm MCE membrane, 47 mm, Merck, GSWP04700, Darmstadt, Germany). Each 2 L replicate was filtered through an individual membrane. To control for possible contamination, MilliQ water was processed through independent filters following the same procedure. Multiple decontamination measures were adopted, including UV light exposure. All filtration equipment (funnels, tweezers, scissors) and the working area were sterilized with 10% bleach followed by 96% ethanol. Following the protocol of Thomsen et al. [39], the filters were cut into 1 mm strips prior to eDNA extraction, which was carried out using the DNeasy Blood & Tissue Kit (Cat no: 69504; Qiagen, Hilden, Germany). Extracted eDNA samples were preserved at −20 °C until further analysis.

### 2.3. eDNA Library Preparation and Bioinformatics Analysis

IGA Technology Services S.r.l. (Udine, Italy) performed the metabarcoding analysis of the eDNA water samples. For this study, we chose 12S primers originally designed by Riaz et al. (2011) [40], which have been widely applied in eDNA studies due to their high specificity for vertebrates, their ability to amplify short fragments suitable for degraded DNA (common in environmental samples), and their demonstrated effectiveness for detecting fish and amphibians in water samples [41,42]. These primers were also validated on a mock community consisting of DNA from 24 vertebrate and invertebrate species, successfully detecting all vertebrates present.

Library preparation consisted of two steps of PCR amplification; the first step was performed with the primers 12SV5-F 5′-ACTGGGATTAGATACCCC-3′ and 12SV5-R 5′- TAGAACAGGCTCCTCTAG-3′ [40]. A total reaction volume of 25 µL was prepared for the PCR, containing 12.5 µL of 2× KAPA HiFi HotStart ReadyMix (Roche, Wilmington, MA, USA), 2.5 µL of the 2 µM forward primer, and 2.5 µL of the 2 µM reverse primer. A total of 50 ng of extracted DNA was added to the PCR mixture before thermal cycling. The amplification protocol involved an initial denaturation step at 95 °C for 3 min, followed by 30 cycles of denaturation at 95 °C for 30 s, annealing at 55 °C for 30 s, and extension at 72 °C for 30 s. A final elongation step was performed at 72 °C for 5 min. The resulting amplicons were purified using Ampure XP beads (1.6× ratio; Beckman Coulter Life Sciences, Indianapolis, IN, USA) and eluted in 35 µL of Tris-HCl buffer (pH 8.0). For the indexing PCR, 7.5 µL of the purified product was mixed with 12.5 µL of 2× KAPA HiFi HotStart ReadyMix (Roche, Wilmington, MA, USA) and 2.5 µL of each Nextera XT index primer (Illumina, San Diego, CA, USA). The thermal profile consisted of an initial denaturation for 3 min at 95 °C, followed by 9 cycles of 30 s at 95 °C, 30 s at 55 °C, and 30 s at 72 °C, concluding with a final extension of 5 min at 72 °C. The concentration of indexed amplicons was measured using the Qubit 1X dsDNA HS Assay Kit (Thermo Fisher Scientific, Waltham, MA, USA). Equimolar amounts of each library were pooled and sequenced on the Illumina MiSeq platform (Illumina, San Diego, CA, USA) using a 2 × 300 bp paired-end configuration. Base calling, demultiplexing, and adapter removal were carried out automatically by the MiSeq Reporter software v4.1.0. Data processing was conducted using an in-house metabarcoding analysis pipeline. When the amplicon length allowed, paired-end reads were merged with FLASH v1.2.11 [43] (parameters: --max-overlap 70 --min-overlap 8) to generate consensus pseudo-reads, while unmerged pairs were retained as independent reads. Both merged and unmerged reads were preserved for further analysis. Primer sequences targeting the 12S variable region were removed using Cutadapt v2.7 [44] (--discard-untrimmed --minimum-length 70 --overlap 10 --times 2 --errorrate 0.15), and reads shorter than 70 bp were discarded. Low-quality bases at the 3′ ends were trimmed with ERNE-FILTER v1.4.3 [45] using the parameter --min-size 70. Subsequently, the QIIME pipeline v1.9.1 [46] was used for downstream processing. Chimera detection was performed with VSEARCH v2.14.1 [47], and Operational Taxonomic Units (OTUs) were identified in “open-reference” mode against the 12S Vertebrate Reference Set for the RDP Classifier (release v2.0.0, available at Zenodo) [48,49,50]. Reference sequences were retrieved from the NCBI nucleotide database (July 2021) and MitoFish (March 2020), comprising 19,654 sequences representing 15,007 taxa, including 9564 species. Taxonomic assignment was conducted using the RDP Classifier v2.2 with predefined taxonomy mapping, based on the NCBI taxonomy database. Only OTUs showing ≥97% identity and ≥0.50 confidence were retained for subsequent analyses.

### 2.4. Data Analysis

Species richness (S) and biodiversity indices (H′) of species identified through eDNA in the two Sicilian lakes were calculated using the Shannon–Wiener index. The species detected in the two lakes were then compared using the Bray–Curtis similarity index, which accounts for both presence–absence and quantitative information on species composition. To account for differences in the number of fragments detected across vertebrate taxa, we applied a post-analysis base-10 logarithmic transformation of fragment counts. To compare the two communities, a PERMANOVA analysis was performed.

## 3. Results

**Figure 2:**
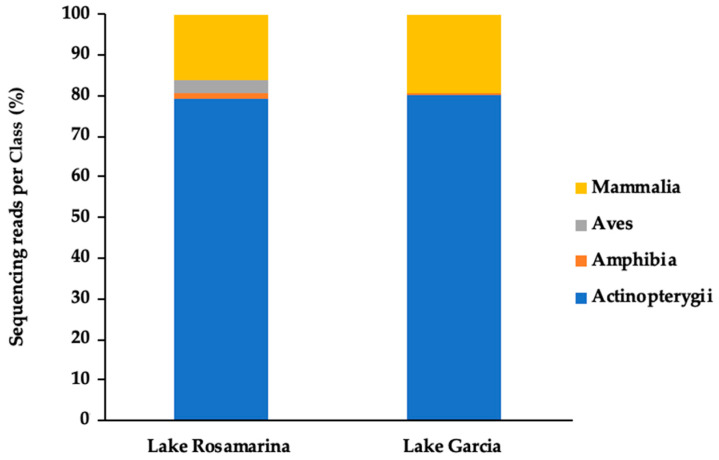
Vertebrate classes detected in Lake Rosamarina and Lake Garcia. Bar charts display the percentage of sequencing reads assigned to each taxonomic Class per sample.

After taxonomic filtering and the removal of human and unclassified sequences, the eDNA analysis yielded a total of 64,205 fragments from Lake Rosamarina and 582,437 from Lake Garcia. At the Class level, the same four taxonomic classes were identified in both lakes (Figure 2). In both sites, the majority of fragments belonged to the Class Actinopterygii (79.5% in Lake Rosamarina; 80.2% in Lake Garcia) and Mammalia (16.1% in Lake Rosamarina; 19.3% in Lake Garcia), while Amphibia and Aves were detected at lower proportions.

**Figure 3:**
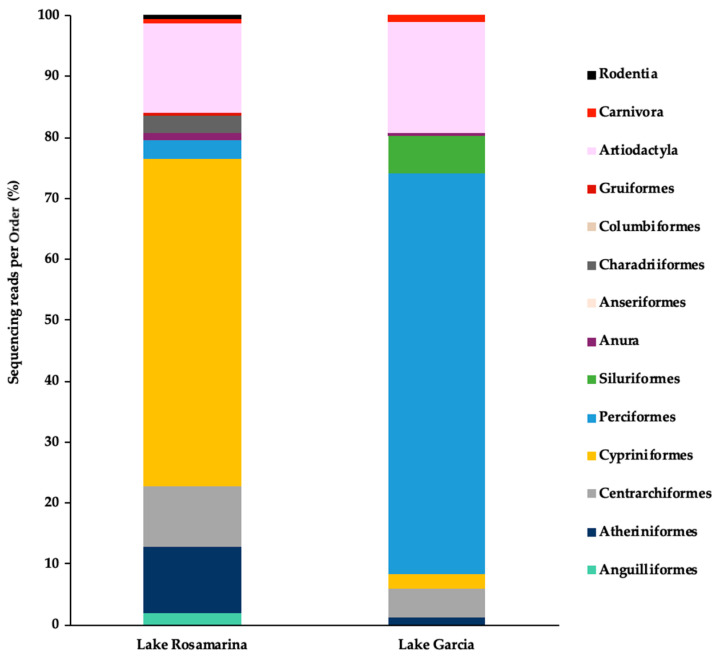
Vertebrate orders detected in Lake Rosamarina and Lake Garcia. Bar charts display the percentage of sequencing reads assigned to each taxonomic Order per sample.

A total of 14 taxonomic orders were identified across the two lakes, with 9 orders detected in Lake Garcia and 13 in Lake Rosamarina (Figure 3). In Lake Garcia, the highest percentage of eDNA fragments belonged to Perciformes (65.9%), whereas in Lake Rosamarina, Cypriniformes accounted for the largest proportion of fragments (53.7%). Eight orders were shared between both lakes: Atheriniformes, Centrarchiformes, Cypriniformes, Perciformes, Anura, Columbiformes, Artiodactyla, and Carnivora, although their percentages of fragments varied. In particular, Artiodactyla fragments were present in both lakes, with substantial proportions (Lake Garcia 18.4%; Lake Rosamarina 14.7%). The Order Siluriformes was detected exclusively in Lake Garcia, at a percentage of 6.1%, while Anguilliformes, Anseriformes, Charadriiformes, Gruiformes, and Rodentia were unique to Lake Rosamarina, each with a percentage below 3%.

**Figure 4:**
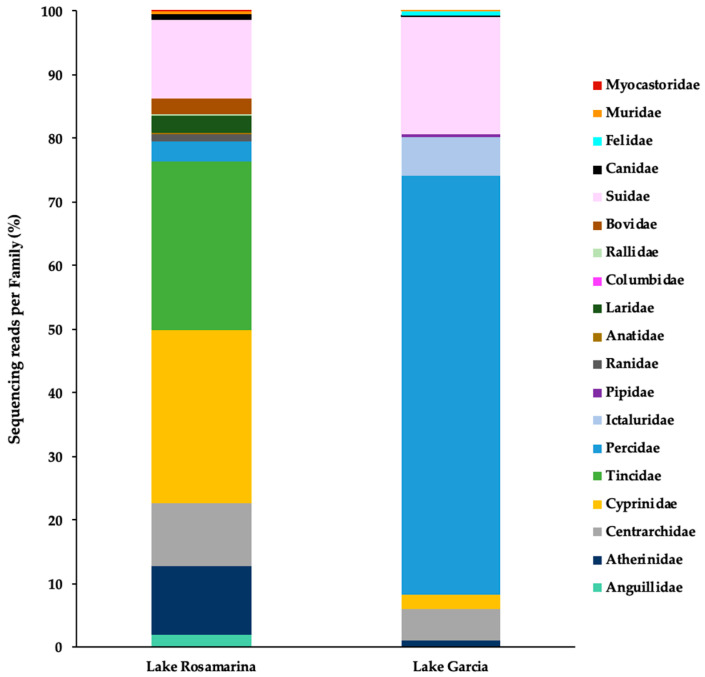
Vertebrate families detected in Lake Rosamarina and Lake Garcia. Bar charts display the percentage of sequencing reads assigned to each taxonomic Family per sample.

A total of 16 families were identified in Lake Rosamarina and 13 in Lake Garcia (Figure 4). In Lake Rosamarina, the highest percentages of eDNA fragments among families were observed for Cyprinidae (27.0%), Tincidae (26.6%), Atherinidae (10.8%), and Centrarchidae (9.9%). Except for the Tincidae, these same families were also present in Lake Garcia, but at significantly lower percentages of fragments (ranging from 4.8% to 1.2%). In contrast, Percidae was the dominant Family in Lake Garcia (65.9%), while in Lake Rosamarina it represented only 3.0%. Several families identified exclusively in Lake Rosamarina included Anguillidae, Ranidae, Anatidae, Laridae, Rallidae, Muridae, and Myocastoridae, with each contributing less than 2.1% of the total reads. Conversely, Ictaluridae (6.1%), Pipidae (0.4%), and Felidae (0.7%) were detected only in Lake Garcia. The Family Suidae was found in both lakes, with notable percentages of eDNA fragments: 12.4% in Lake Rosamarina and 18.3% in Lake Garcia. Additionally, Columbidae, Bovidae, and Canidae were identified in both sites, although each accounted for less than 2.3% of the total fragments.

**Figure 5:**
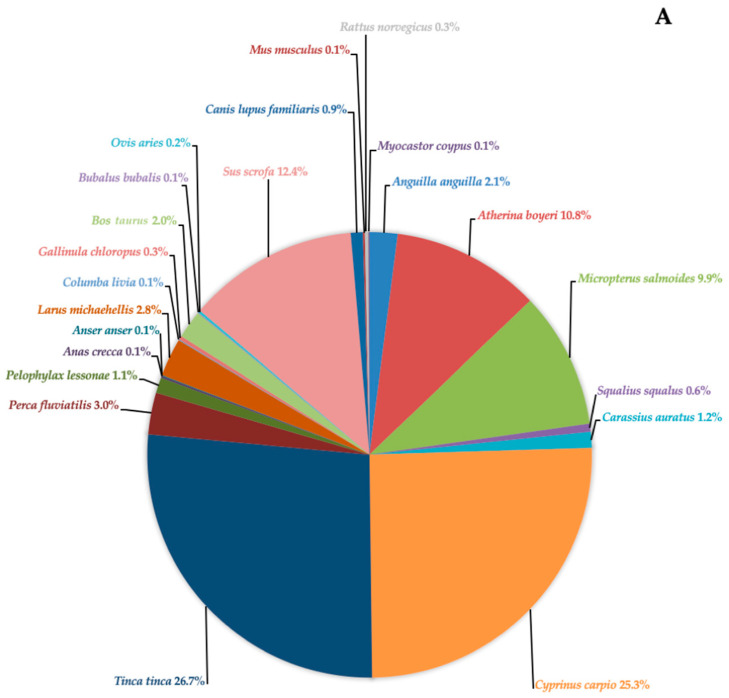

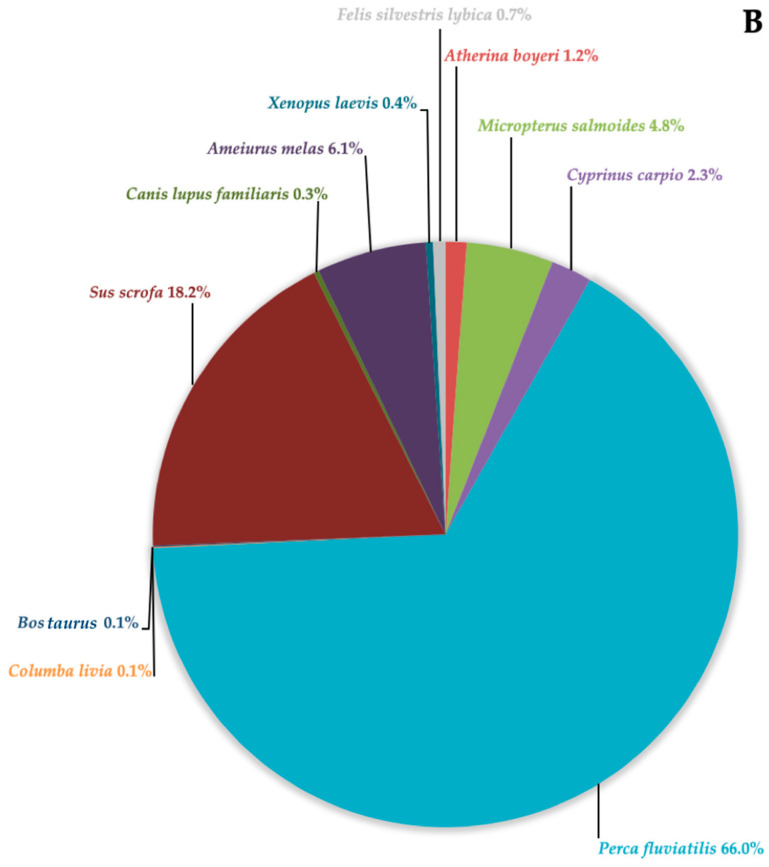
Pie chart (expressed as a percentage) representing the species identified in Lake Rosamarina (**A**) and Lake Garcia (**B**) using eDNA analysis. Percentages indicate the proportion of DNA fragments found per species across the collected samples.

The eDNA analysis revealed a total of 22 taxa, both aquatic and terrestrial, in Lake Rosamarina (Figure 5A). Among these, native species such as *Anguilla anguilla*, *Atherina boyeri*, *Anas crecca*, *Anser anser*, *Larus michahellis*, and *Gallinula chloropus* were detected, together with non-native but naturalized taxa like *Tinca tinca* and *Cyprinus carpio*. Several allochthonous and invasive species were also identified, including *Micropterus salmoides*, *Squalius squalus*, *Carassius auratus*, *Perca fluviatilis*, and the semi-aquatic rodent *Myocastor coypus*. The most frequently detected species at this site were *T. tinca* (26.7%), *C. carpio* (25.3%), *M. salmoides* (9.9%), *Sus scrofa* (12.4%), and *A. boyeri* (10.8%). Other species were detected with a frequency between 0.05% and 3% and are shown in Figure 5A. In Lake Garcia (Figure 5B), 11 taxa, both aquatic and terrestrial, were identified. The vertebrate community was dominated by invasive species such as *P. fluviatilis* (65.9%), followed by *S. scrofa* (18.15%), and several other non-native and invasive species such as *Ameiurus melas* (6.1%), *M. salmoides* (4.8%), and *C. carpio* (2.3%). Also detected were *Felis silvestris lybica*, *Columba livia*, domestic species such as *Bos taurus* and *Canis lupus familiaris*, further native species like *A. boyeri* and the invasive amphibian *Xenopus laevis*. A total of eight species were shared between the two lakes: *A. boyeri*, *M. salmoides*, *C. carpio*, *P. fluviatilis*, *B. taurus*, *S. scrofa*, *C. livia*, and *C. l. familiaris*.

**Figure 6:**
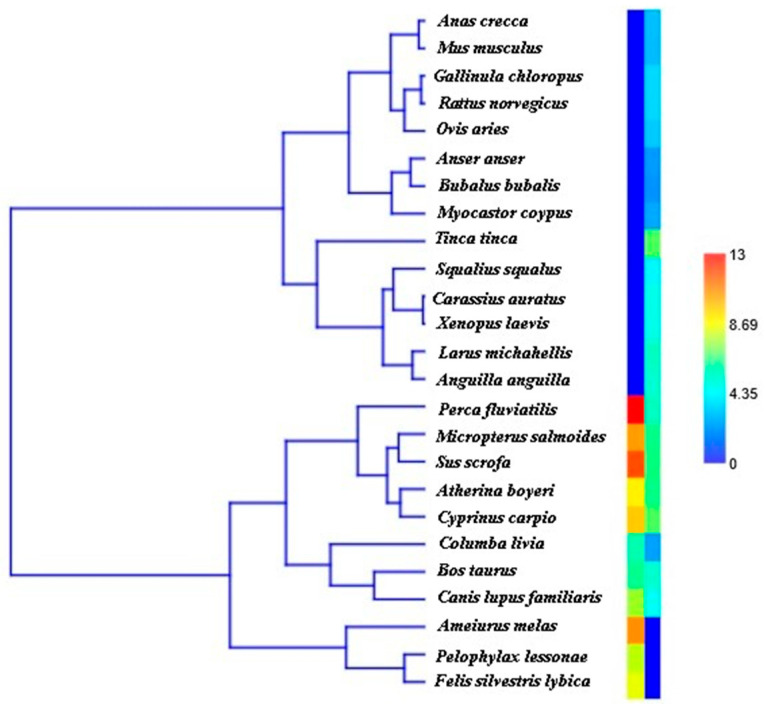
Dendrogram of the Bray–Curtis quali-quantitative similarity between species from Lake Rosamarina and Garcia, using the UPGMA method (left); dendrogram and heatmap of the Bray–Curtis quali-quantitative similarity at the species level of the Sicilian lakes using the UPGMA method (right). The heatmap visually represents species across samples, using color gradients to indicate variations in distribution levels, with red indicating the highest, blue representing the lowest, and green depicting intermediate levels.

To support the previous findings, a hierarchical clustering analysis combined with a heatmap was performed to examine the faunal communities detected in Lake Garcia and Lake Rosamarina (Figure 6). The dendrogram on the right illustrates the similarity relationships among the identified species based on their distribution across the two lakes. The accompanying heatmap shows species presence and quantitative information on species composition, with color intensity ranging from blue (low) to red (high), as indicated by the sidebar scale. Each row represents a species, while columns correspond to the two lakes. The analysis reveals a clear differentiation in the faunal composition between the two lakes, suggesting the presence of distinct ecological communities. The taxonomic diversity observed—including mammals, birds, fish, reptiles, and amphibians—indicates a high level of ecological heterogeneity across the two environments. Furthermore, to minimize the effect of random variability, the logarithms of fragment counts were used in the analyses, making the values more comparable.

Despite the fact that the surface areas of the two lakes are almost identical, species richness and diversity (Table 1) were significantly lower in Lake Garcia compared to Lake Rosamarina. The entire community recorded in the former reservoir appears to represent only a fraction of that found in the latter.

PERMANOVA analysis based on Bray–Curtis distances between the communities revealed a significant difference in species composition between the two sites (pseudo-F = 5.4, *p* = 0.001, 999 permutations). The two communities are clearly distinct: Garcia hosts taxa absent from Rosamarina (e.g., *Ameiurus melas*, *Felis silvestris lybica*, *Xenopus laevis*), while Rosamarina includes species not detected in Garcia (e.g., *Larus michahellis*, *Carassius auratus*, *Pelophylax lessonae*, *Myocastor coypus*). Some taxa are shared between the two lakes but occur at different relative abundances (e.g., *Perca fluviatilis*, 12.08% vs. 5.05%; *Rutilus rutilus*, 2.47% vs. 6.49%). These results confirm that the biological communities of Garcia and Rosamarina do not overlap but instead show marked differences in both structure and composition.

## 4. Discussion

The Mediterranean region is considered one of the main biodiversity hotspots in the world, characterized by a high number of endemic and rare taxa and, at the same time, subject to strong anthropogenic pressures that are currently increasing the loss of biodiversity as well as the speed with which this loss is occurring [51]. Given the current poor and incomplete knowledge of the biodiversity of these areas, understanding which species are present and their spatial-temporal distribution in these environments becomes essential for evaluating the ecological status and dynamics of aquatic ecosystems [52]. Until now, traditional monitoring methods based, for example, on direct capture or visual observation have been used to census biodiversity, providing irreplaceable morphological and ecological information. However, these techniques, in addition to their notable advantages, present many logistical and operational limitations that are becoming increasingly important to overcome: high costs in terms of time, resources, and skills required, and limited effectiveness in the detection of rare, elusive, or low-density vertebrate species. In light of this, the monitoring of aquatic biodiversity increasingly requires innovative approaches capable of overcoming these constraints [53]. In this context, DNA metabarcoding, and, in particular, eDNA analysis, is a new tool to complement traditional methods and that can provide information on biodiversity in a non-invasive, rapid, and efficient manner [54,55].

In this pilot study, eDNA analysis was applied to provide a first snapshot of vertebrate biodiversity in two lakes of western Sicily: Lake Rosamarina and Lake Garcia. The aim was to expand our knowledge of vertebrate biodiversity in Sicilian inland waters, thus furthering the aim of a much larger project concerning the creation of a first baseline of inland waters in the province of Palermo by implementing the initial data already collected by Mauro et al. [37,38], who first used eDNA to assess vertebrate and invertebrate communities in three Sicilian lakes: Poma, Piana degli Albanesi and Scanzano. By extending the geographical and ecological coverage to two additional basins, our study contributes to developing a broader and more robust reference base for the biodiversity of Sicilian freshwater ecosystems. The results of this study revealed a notable difference in the variety of vertebrates detected between the two basins; in fact, Lake Rosamarina exhibited higher taxonomic richness (classes, orders, families, and species) than Lake Garcia.

The observed differences in biodiversity between the two lakes could reflect real envi-ronmental variation, although they could also result from heterogeneous distribution or degradation of eDNA. Indeed, the persistence and detectability of eDNA in aquatic systems are influenced by environmental factors such as temperature, pH, and water flow [56,57,58]. Furthermore, because taxa release eDNA in different quantities and at varying rates, eDNA analyses cannot fully and accurately reflect the true relative abundance of species [59,60]. For this reason, the number of fragments assigned to in-dividual taxa was used as an indirect proxy for relative abundance, and a logarithmic transformation of the abundance data was applied to reduce variability and improve comparability. Despite these limitations, eDNA analysis has proven effective and versatile, allowing the simultaneous detection of aquatic, semi-aquatic, and terrestrial taxa and providing a overview of local biodiversity. In both lakes, native and non-native taxa were found among the aquatic species identified, some of which are potentially invasive. Lake Rosamarina showed a richer community compared to Lake Garcia, with strictly aquatic species identified in it. Among them, *T. tinca* and *C. carpio* were the most frequently detected. *T. tinca* is a species widely reported in the region [61]; although it is native to a large area of Eurasia, in many areas, including Sicily, *T. tinca* has been introduced by man (e.g., for fishing or aquaculture). In Sicily, Tortonese (1970) [62] regarded the species as native, whereas Vinciguerra (1896) [63] suggested it was introduced by the Normans. More recent studies, however, classify *T. tinca* as a species which was introduced to the island [9,61]. To our knowledge, no previous study has documented its presence in Lake Rosamarina using traditional methods. Nonetheless, its occurrence in Sicily is well established: Ferrito and Tigano (1995) [64] reported it in the Simeto River; Duchi (2006) [65] reported it in the Irminio and Tellaro rivers, as well as in its tributary, the Tellesimo; Duchi and Milano (2014) [66] recorded it in Lake Lentini through preliminary surveys and fishing activities, and Duchi and Divincenzo (2017) [67] documented it in the Sant’Elia stream. This species, typical of calm, stagnant, or slow-flowing waters with muddy bottoms and rich in submerged vegetation, can be considered a potential bioindicator of poor water quality; its remarkable resistance to adverse environmental conditions makes it suitable for studies on the accumulation of pollutants (e.g., heavy metals or pesticides) in tissues, providing indications on water contamination [68]. Studies have demonstrated its usefulness in biomonitoring contamination by carbofuran (a pesticide) and lead [69,70,71,72]. In addition, its presence, especially in conjunction with other non-native species, can be associated with eutrophication processes contributing to the shift in lakes and ponds from clear waters rich in macrophytes to turbid and algal water bodies [66].

*C. carpio* is a Eurasian cyprinid which has been widely introduced into Italy for food and recreational purposes [73]. This species was also detected in our previous study in two other lakes in the province of Palermo using eDNA analysis, Lake Piana (47%) and Lake Scanzano (0.16%), representing one of the most frequently recorded aquatic species in Lake Piana [37] and in the Fosso del Tempio river system in Sicily through eDNA surveys [74]. It has also been reported through traditional methods in the Irminio and Tellaro Rivers, and the Gariffi basin [65]. In addition, *C. carpio* had already been documented in Lake Rosamarina and several other Sicilian lakes by Duchi and Milano (2014) [65] through field surveys and preliminary catches. Further records include Lago Preola and Gorghi Tondi, a nature reserve where four individuals of *C. carpio* were also captured in fyke nets during turtle sampling [75]. The carp is an omnivorous species which feeds by digging into sediments to search for invertebrates, debris, and plant material: this behavior causes bioturbation, increasing the turbidity of the water and reducing the submerged plant cover with negative impacts on aquatic biodiversity. It is also a eurythermal species, so climate change could expand its invasive potential, making some areas more favorable to its proliferation. Like *T. tinca*, the common carp is also associated with eutrophic ecosystems, with high turbidity, and low water quality.

Another frequently detected species was *M. salmoides*, a North American predatory species introduced for sport fishing. This species can have significant impacts on native freshwater ecosystems and is frequently reported in Sicilian inland waters [9,38]. This species was detected with a high percentage of sequence reads in two lakes in our previous study using eDNA analysis, Lake Scanzano (23%) and Lake Poma (43%), in which it was the most frequently recorded aquatic species [38]. It was also reported through traditional survey techniques by Duchi (2006) [65], who recorded its presence in the Irminio and Tellaro rivers, and by Duchi and Milano (2014) [66] who documented it in several other Sicilian lakes, including Ogliastro, San Giovanni, Nicoletti, Piana degli Albanesi, Ancipa, and Arancio. The species is also included in the checklist of alien species present in Sicily [9]. Recent studies have shown that *M. salmoides* modifies its diet based on habitat complexity, a trophic plasticity that makes it a highly adaptable and competitive predator, leading to the decline of native species due to predation and competition [76]. For this reason, it is considered one of the 100 worst invasive species in the world by the IUCN. Its dominance often indicates ecological simplification as it is associated with ecosystems which exhibit low structural complexity and reduced fish diversity [77]. Its massive presence may therefore signal a trophic imbalance and a need for management interventions. Compared to tench and carp, whose ecological role as an indicator of chemical pollution has already been discussed, *M. salmoides* is more of a bioindicator of ecological alterations [78].

In addition to *M. salmoides*, another bioindicator of ecological imbalance is *P. fluviatilis*, a potentially invasive predatory fish whose presence in Italy and Sicily has been documented for centuries [9,79] and was confirmed by the high number of eDNA fragments detected in our previous study in Lake Poma (31%) and Lake Piana (47%) [38].

Its presence had already been reported in Lake Rosamarina, and it is widely distributed across Sicilian lakes—including Piana degli Albanesi, Ancipa, Poma, Pozzillo, and Trinità—based on direct captures or fishermen’s reports [66]. Moreover, *P. fluviatilis* is also included in the checklist of non-native species present in Sicily [9]. *P. fluviatilis* is also an extremely eurivalent species [80] native to parts of Great Britain, northern Europe, and Asia [81,82]; it has been introduced intentionally or unintentionally into many other parts of the world, from China [83,84], to Australia [85], Portugal [86], Corsica [87], and Italy [88].

To date, no studies have attempted to directly compare the impacts of European perch on water quality but, as a juvenile predator of large filter-feeding cladocerans, it would appear to be a potential contributor to water turbidity, allowing for the increase in planktonic algae [89,90].

Other fish species recorded in Lake Rosamarina include *C. auratus*, an ornamental Asian species often released into the wild [91]. It was the most frequently detected species in our previous study using eDNA analysis in Lake Scanzano (75%) [37]. The wide distribution of this species in Sicilian lakes has been well documented also by traditional methods: it has long been recorded in Sicily, for example, in the Simeto river [64], as well as in rivers of the Ragusa area such as the Irminio and Tellaro [65], and in Lake Urio Quattrocchi [92]. In addition, Duchi and Milano (2014) [66] reported its presence in Lake Rosamarina, but also in many other Sicilian lakes such as Lentini, Ogliastro, San Giovanni, Nicoletti, Piana degli Albanesi, Arancio, Santa Rosalia, Pozzillo, and Dirillo. The success of this species can be explained by the fact that it survives under hypoxic and even prolonged anoxic conditions thanks to a highly efficient anaerobic metabolism; similarly, it resists strong variations in temperature, pH, and turbidity, which makes it potentially invasive in different habitats [92]. This means that monitoring this species is essential for the protection of natural habitats. *C. auratus* is also widely used in ecotoxicology to test the sublethal effects of heavy metals, pesticides, and microplastics, as well as physiological responses to environmental stress; however, its high tolerance makes it more suitable for studies on stress resistance than for early indications of environmental degradation.

Another species found in our study was *A. boyeri*, a euryhaline fish capable of adapting to varying salinity levels, commonly found in coastal and lacustrine environments of Sicily [93]. It had been previously detected in Lake Poma (16%) and Lake Scanzano (0.72%) in our earlier study [37], and had already been reported in Lake Rosamarina through captures [66]. Moreover, this species has also been recorded in other Sicilian freshwater contexts, particularly in the Dirillo and Irminio Rivers, as well as in several coastal ponds [65].

*A. boyeri* inhabits unstable and variable environments such as lagoons and estuaries, where salinity, temperature, and oxygen can change rapidly [94]. Studies in the Caspian Sea have shown cardiac alterations in response to polycyclic aromatic hydrocarbons (PAH), suggesting its use as a bioindicator of pollution [95]. Unlike the previously discussed species, its sensitivity to sublethal contaminants and its constant presence in transitional environments make it useful for ecotoxicological monitoring. Caliani et al. (2019) [96], for example, highlighted the possibility of using ecotoxicological biomarkers of *A. boyeri* to reveal potential contamination in the transitional aquatic ecosystem at Capo Peloro (north-eastern Sicily), thus demonstrating the sensitivity of the species and its possible use as a bioindicator.

*Squalius squalus*, a cyprinid native to central and northern Italy, was also identified; its presence in Sicily is attributed to introduction activities [97]. According to Kottelat and Freyhof (2007) [98], the species may be present in Sicily, but the record is not confirmed (Duchi, pers. comm.). Likewise, CK2000 [99] suggested that it was introduced in Sicily in recent years, so its presence in Lake Rosamarina cannot be completely excluded.

*S. squalus* can also be considered a bioindicator for several aspects of the environmental health of water courses: its remarkable tolerance of different environmental conditions, including some levels of pollution, makes it an indicator of environmental degradation. Studies have highlighted its greater tolerance of chemical pollutants compared to other fish and its ability to accumulate contaminants [100,101,102,103].

Finally, among the Actinopterygii, a particularly notable finding was the detection of *A. anguilla*, a native European species listed as “Critically Endangered” on the IUCN Red List [104,105] due to artificial barriers (dams, barrages), but also to pollution and loss of habitat, as well as fishing and illegal trade. This finding underscores the potential importance of Mediterranean lake environments as refuges for threatened taxa. In our previous study using eDNA, we did not detect this species; however, using traditional methods, it has been found in Sicilian lakes including Rosamarina, as well as Lentini Ogliastro, Santa Rosalia, and Dirillo [66]. Additional records also exist from other basins in southern Sicily, such as the Rifriscolaro and Favara streams, the Irminio and Tellaro Rivers, and the latter’s tributary the Tellesimo, as well as several coastal ponds [65].

It can live in fresh, brackish, and marine waters, with great adaptability to variable chemical-physical conditions and, being a catadromous species, it works as an ecological connector between marine and continental environments, contributing to the flow of energy and nutrients. Although it is a euryhaline species, compared to the other fish found by our analyses in Lake Rosamarina, *A. anguilla* is sensitive to heavy metals and organic pollutants, so its presence could partly indicate an ecosystem that is not completely degraded.

Moreover, the use of eDNA allowed for the identification of amphibian species such as *Pelophylax lessonae*, previously reported in Sicily [105]. Compared to most of the aquatic species detected by our analyses and already discussed, *P. lessonae* is linked to well-preserved ecosystems and is highly sensitive to pollution; Dufresnes et al. [106] used the eDNA technique to monitor this species, whose presence in Western Europe is compromised by biological invasions.

In Lake Garcia, six strictly aquatic species were identified, among which *P. fluviatilis* was found in higher percentages of fragments. To our knowledge, no other scientific studies have reported this species in this lake using either traditional methods or eDNA analysis; however, as mentioned above, it has been detected in other Sicilian lakes using traditional methods [66]. Several non-native species already identified in Lake Rosamarina were also found, including *M. salmoides*, *C. carpio*, and *A. boyeri*. For the first two species, their presence had already been reported in these and other Sicilian lakes using traditional methods by Duchi and Milano [66]. The presence of these species in both basins suggests a high degree of adaptability and successful establishment. In addition, the presence of *A. melas*, a North American species previously reported in Sicily, was detected. This species has become naturalized in numerous Sicilian freshwater environments due to its high tolerance of variable environmental conditions [9], and it was detected in Lake Poma (10%) in our previous study [37]. Furthermore, particularly noteworthy was the identification of *X. laevis*, an invasive frog species whose presence in Sicily has been documented since 2004 [107]. The Sicilian population is currently considered one of the largest and densest in Europe [108]. The detection of *X. laevis* is ecologically significant as this species is known for its negative impact on aquatic ecosystems: its diet includes aquatic larvae of nectonic insects, small planktonic crustaceans, as well as the eggs and larvae of native amphibians [109]. These feeding habits, combined with its ability to act as a vector of pathogens, can contribute to a reduction in local biodiversity and compromise the ecological stability of invaded habitats [110].

The eDNA approach enabled the detection of species that are not strictly aquatic, yet still dependent to some extent on these ecosystems. In Lake Rosamarina, several bird species were identified, including *A. crecca*, *A. anser*, and *G. chloropus*, as well as *C. livia* and *L. michahellis*. Notably, the latter two are opportunistic and synanthropic species whose presence suggests frequent interactions between wildlife and human activities in the area. Unfortunately, no other systematic studies based on traditional monitoring methods are available for the two basins investigated; only occasional reports of fauna species exist. In both basins, several domestic terrestrial species were identified: *C. l. familiaris*, *F. s. lybica*, *B. taurus*, *Ovis aries*, *Bubalus bubalis* and *S. scrofa*. Most of them were also detected in our previous study in Lake Poma (*C. l. familiaris* 84%, *S. scrofa* 3%), Lake Scanzano (*C. l. familiaris* 0.1%, *O. aries* 7%, *S. scrofa* 93%), and Lake Piana (*C. l. familiaris* 73%, *B. taurus* 3%, *O. aries* 1%, *S. scrofa* 6%) [37]. The detection of these species via eDNA was likely due to the usage dynamics of surrounding areas. In particular, the presence of *B. taurus*, *O. aries* and *C. l. familiaris* could be connected to the widespread practice among local shepherds of leading domestic animals near the reservoirs for watering, thus favoring the release of genetic material into the aquatic environment. As for *S. scrofa* and *F. s. lybica*, their identification can be traced back to synanthropic or semi-wild populations present in the area whose DNA could be transported into the basins through surface streams, small tributaries, or directly by contact with water. Finally, the presence of the eDNA of domestic species could also derive from anthropogenic and agricultural activities in the surroundings of the lakes which facilitate the transport of biological residues through soil leaching or drainage channels flowing directly into water bodies. These results highlight the interconnection between the terrestrial and aquatic environments and underline the usefulness of eDNA as a tool to monitor not only aquatic biodiversity, but also the impact of human activities on lake ecosystems [111,112,113]. In addition to the domestic terrestrial species, three wild terrestrial species have been identified exclusively in Lake Rosamarina: *Rattus norvegicus*, *Mus musculus* and *M. coypus*. The latter is an invasive species, native to South America, whose negative impact on marsh habitats and embankments is well documented [114,115]. The introduction of this species into Sicily is believed to date back to the 1990s [116], with a very small population, concentrated and localized in a specific area of southeastern Sicily, about which no further evidence is currently available [117]. Given the distance, approximately 150 km in a straight line, it is highly unlikely that its presence in Lake Rosamarina is connected to that population. A verification of its actual presence in the lake, a wetland habitat suitable for the species’ ecology, would be necessary.

## 5. Conclusions

The results obtained demonstrate how the eDNA-based approach is able to provide a picture of vertebrate biodiversity in lake environments, highlighting the coexistence of native, alien, and invasive species, as well as the influence of human activities on freshwater ecosystems. This information marks a fundamental starting point for future conservation, monitoring, and sustainable management of biodiversity in Sicilian inland waters.

Lake Rosamarina showed greater taxonomic richness than Lake Garcia, particularly in aquatic and semi-aquatic species. These findings underscore the need for concrete conservation and management actions in Sicilian freshwater ecosystems. On the other hand, the low biodiversity recorded in Lake Garcia highlights the importance of habitat restoration measures, such as enhancing aquatic vegetation coverage. Invasive species like *M. salmoides* and *X. laevis* were detected, which could necessitate a combined prevention and control strategy, including regular monitoring (e.g., quarterly eDNA sampling) and physical removal through targeted fishing.

In conclusion, our results highlight the potential of the eDNA approach as a sensitive and effective tool for studying biodiversity in these ecosystems. On the other hand, to obtain a more comprehensive picture, the data should be integrated with traditional sampling methods and morphological identification. We acknowledge that assessing biodiversity at a single time point represents a limitation, as it provides only a “snapshot” of the ecosystem and does not capture seasonal or temporal variations. Nevertheless, the aim of this study was to provide an initial assessment of vertebrate biodiversity and to establish a standardized methodology for future surveys. Therefore, conducting seasonal and repeated surveys is a vital step toward a more comprehensive understanding of aquatic community dynamics and toward distinguish transient indicators from stable ecological patterns. In the future, we would like explore the correlation between environmental factors (such as water temperature, pH, nutrient concentration, habitat complexity, etc.) and biodiversity. These analyses could identify the key drivers of community differences, strengthening the relevance of the research findings for ecological management. Future research should build on this work to address temporal dynamics and provide a more complete and representative understanding of ecosystem biodiversity. Looking forward, the application of eDNA technology can be further enhanced by developing specific primers tailored to Sicilian freshwater ecosystems and by constructing a regional DNA barcode database through high-throughput sequencing. These efforts would not only improve biodiversity monitoring but also increase the predictive and applied value of future research in the region. Moreover, future study could consider alternative strategies, such as using multiple primer sets targeting complementary taxonomic groups, or applying statistical models that account for technical and biological sources of variation. These approaches, although more complex, represent promising directions for improving the robustness of quantitative community profiling in eDNA-based studies.

## Figures and Tables

**Table 1:** Comparison between the number of taxa and the values of the biodiversity index in the sample. The richness value (taxa_S) and the biodiversity indices were calculated with the Shannon (H) algorithm.

	Lake Rosamarina	Lake Garcia
Taxa_S	24	16
Shannon_H	3.148	2.419

## Data Availability

Non-public data for privacy, contact the authors.
